# The Hemocompatibility of Nanoparticles: A Review of Cell–Nanoparticle Interactions and Hemostasis

**DOI:** 10.3390/cells8101209

**Published:** 2019-10-07

**Authors:** Kara M. de la Harpe, Pierre P.D. Kondiah, Yahya E. Choonara, Thashree Marimuthu, Lisa C. du Toit, Viness Pillay

**Affiliations:** Wits Advanced Drug Delivery Platform Research Unit, Department of Pharmacy and Pharmacology, School of Therapeutic Science, Faculty of Health Sciences, University of the Witwatersrand, Johannesburg, 7 York Road, Parktown 2193, South Africa; karadelaharpe7@gmail.com (K.M.d.l.H.); yahya.choonara@wits.ac.za (Y.E.C.); thashree.marimuthu@wits.ac.za (T.M.); lisa.dutoit1@wits.ac.za (L.C.d.T.)

**Keywords:** hemocompatibility, nanoparticles, erythrocytes, platelets, leukocytes

## Abstract

Understanding cell–nanoparticle interactions is critical to developing effective nanosized drug delivery systems. Nanoparticles have already advanced the treatment of several challenging conditions including cancer and human immunodeficiency virus (HIV), yet still hold the potential to improve drug delivery to elusive target sites. Even though most nanoparticles will encounter blood at a certain stage of their transport through the body, the interactions between nanoparticles and blood cells is still poorly understood and the importance of evaluating nanoparticle hemocompatibility is vastly understated. In contrast to most review articles that look at the interference of nanoparticles with the intricate coagulation cascade, this review will explore nanoparticle hemocompatibility from a cellular angle. The most important functions of the three cellular components of blood, namely erythrocytes, platelets and leukocytes, in hemostasis are highlighted. The potential deleterious effects that nanoparticles can have on these cells are discussed and insight is provided into some of the complex mechanisms involved in nanoparticle–blood cell interactions. Throughout the review, emphasis is placed on the importance of undertaking thorough, all-inclusive hemocompatibility studies on newly engineered nanoparticles to facilitate their translation into clinical application.

## 1. Introduction

Blood is not only the first contact for nanoparticles (NPs) administered intravenously, but also the gateway for all NPs, administered via other routes, to reach their target tissues or organs. The size of NPs allows them to easily distribute throughout the body, traverse biological barriers and enter the systemic circulation where they can readily penetrate cells [1]. The size of NPs also makes them more biologically active than micro-sized particles, allowing disruption of the normal cellular biochemical environment. NP interactions with blood components is, therefore, not only inevitable but also potentially perilous and hemocompatibility should be one of the foremost concerns in the design and development of NPs with therapeutic applications [2].

The moment NPs reach the blood system they come into direct contact with blood cells, endothelial cells and plasma proteins, where they can affect the intricate structure and critical functions of these blood components. Plasma proteins instantly adsorb to the surface of NPs to form a protein corona that significantly influences their interaction with blood components and may even lead to increased cellular activation [3]. Recently, NP-induced coagulopathy has become a serious concern with several studies reporting an increased risk of cardiovascular disease due to NP-induced thrombotic complications. Different studies have found that NPs can perturb the coagulation system and cause a shift in the hemostatic balance, resulting in serious life-threatening conditions such as deep vein thrombosis (DVT) and disseminated intravascular coagulopathy (DIC) [4]. The exact mechanisms behind such toxicities have not yet been clearly defined, even though some progress has been made on critical factors that drive the adverse effects of NPs on the hemostatic system. It is important to note that individual NPs have a unique effect on the blood components with even small changes in the composition leading to different mechanisms of interactions and alternative toxicity profiles [5]. 

The most common NPs encountered are carbon-based NPs (fullerenes and carbon nanotubes), metal NPs, ceramic NPs, semiconductors (quantum dots), polymeric NPs and lipid-based NPs [6]. Each constitute unique physiochemical properties that make them indispensable within their fields of application. New and innovative NPs are continuously engineered and have the potential to transform the diagnosis, prevention and treatment of difficult-to-treat conditions such as cancer, Alzheimer’s disease and stroke [7,8,9]. However, very few of these engineered NPs are translated into clinical practice with unforeseen toxicities or unknown cell–NP interactions serving as a barrier to entry. 

Hemocompatibility testing refers to the evaluation of critical interactions between foreign materials and the different components of blood to determine if any adverse effects may arise from the exposure of these foreign materials to blood [10]. The main cellular constituents of blood are the red blood cells (erythrocytes), white blood cells (leukocytes) and platelets (thrombocytes). Each of these blood cells has an intricate physical structure and chemical machinery that allows them to expertly perform their crucial functions in normal hemostasis [11]. As previously mentioned, NPs can easily access these cells and influence both their structure and function that can result in potentially toxic effects. Therefore, researchers should make every effort to conduct thorough hemocompatibility studies on newly engineered NPs that evaluate the interactions between the NPs and all three cellular constituents of blood. This will not only lead to NPs with superior hemocompatibility but can also simplify clinical trials that may follow and fast-track the process of translating newly formulated NP-based products to the market. 

## 2. Erythrocyte Function in Hemostasis and the Mechanisms Involved in Nanoparticle Hemocompatibility

Historically, the role of erythrocytes in hemostasis was neglected and pushed aside as unimportant by researchers. However, clinical evidence argues differently and indicates that erythrocytes have a critical role in normal vascular functioning. For example, a large study with a population of more than 8000 subjects found the incidence of cardiovascular disease (CVD) to be double in the high-hematocrit group compared to the low-hematocrit group, clearly demonstrating the importance of erythrocyte count in normal hemostasis [12]. Such observations have caused researchers to delve deeper into understanding the exact mechanisms whereby erythrocytes influence hemostasis and thrombosis. Through their efforts, interesting discoveries such as the activation of factor IX by the erythrocyte membrane as a possible explanation for its procoagulant activity were made [13]. Currently, erythrocytes are acknowledged as significant to hemostasis and are even being exploited as novel therapeutic targets in thrombotic disorders [14]. An in-depth review of the exact mechanisms whereby erythrocytes contribute to hemostasis was previously published [15] and only gaps in the mechanisms relevant to NP hemocompatibility is discussed in this review. 

Due to their large size and abundance in the blood, erythrocytes mainly contribute to hemostasis and thrombosis through their rheological properties [16]. Hemorheology refers to the influence of blood flow on hemostasis and is largely dependent on blood viscosity and shear rates [17]. Erythrocytes are not only the principal contributors to blood volume and the main determinant of blood viscosity, but they also have distinct flow-affecting properties that give blood the ability to act as a non-Newtonian fluid [18]. Erythrocytes have the tendency to move down the center of the blood vessel, pushing platelets towards the periphery, where they can easily interact with the vasculature to form a hemostatic plug [19]. This phenomenon, called axial margination, is dependent on blood viscosity and can influence the distribution of platelets and NPs within the vasculature. An increase in blood viscosity will enhance platelet margination which intensifies the collisions between the platelets and the vascular wall, thereby increasing the risk of thrombus formation [20]. Axial margination of NPs on the other hand, holds significant implications for the therapeutic efficacy of several nano-scale drug delivery technologies that rely heavily on such margination to reach their therapeutic targets (i.e., the endothelial cells or tissues beyond) [21]. 

There are three main factors that can influence blood viscosity, namely hematocrit, erythrocyte aggregation and erythrocyte deformation [22]. All three factors can be altered by NP–erythrocyte interactions, which underlines the importance of carefully studying the influence of NPs on the critical functions of blood cells.

### 2.1. The Influence of Hematocrit on Blood Viscosity and Hemostasis in Connection with Nanoparticle–Erythrocyte Interactions

Hematocrit refers to the percentage erythrocytes as a function of blood volume and causes an exponential increase in blood viscosity in large blood vessels compared to a linear increase in small vessels where erythrocytes can only pass through in single-file [14]. Hematocrit is directly influenced by hemolysis (i.e., the abnormal rupture or destruction of erythrocytes). Induced hemolysis will lower the hematocrit and has been linked to several fatal clinical conditions such as disseminated intravascular coagulation (DIC) and sickle cell disease (SCD) [23]. 

In addition to altering the hematocrit and hemorheology, a few other mechanisms have been suggested whereby hemolysis can contribute to thrombosis, including the release of erythrocyte-derived macrovesicles, activation of the complement cascade and release of free hemoglobin and heme into circulation which sequesters nitric oxide [24]. Hemolysis is a fundamental parameter in the hemocompatibility testing of biomaterials and can hold significant implications for the clinical use thereof [25]. Numerous NPs including amorphous silica, tricalcium phosphate (TCP), hydroxyapatite (HAP) and especially silver (Ag) NPs have been found to cause significant hemolysis, threatening their use in biomedical applications [26].

The method whereby hemolysis yields adverse effects on hemostasis is prompt and clearly defined, however the methods whereby NPs interact with erythrocytes to cause hemolysis has not been fully elucidated. Different mechanisms have been suggested, including interactions with the erythrocyte membrane, cellular uptake, internalization and oxidative stress. The most prominent mechanism is the direct interaction of NPs with the erythrocyte membrane which can lead to injury, detrimental morphological changes and cytoskeletal distortions [27]. In the case of Ag-NPs, their hemolytic activity is mainly attributed to direct NP–cellular interactions where the particles bind to thiol groups of biological moieties such as proteins and phospholipids in the erythrocyte membrane, leading to denaturation and impaired membrane functioning. Additionally, the negative charge on surface functionalized Ag-NPs will have strong interactions with biological cations in the erythrocyte membrane, further contributing to hemolysis [28]. 

The hemolytic activity of most NPs is concentration-, structure-, size- and shape-dependent. Silica NP toxicity, for example, is directly proportional to the amount of reactive silanol groups exposed on the NP surface which depends on both the size and geometry of the NP [29]. According to Shang et al., size is however the most important of these factors and the sole determinant of effective NP uptake by erythrocytes. Different studies have also found smaller NPs, including gold (Au), titanium dioxide (TiO_2_), and hydroxyapatite NPs, to be more hemolytic than larger particles [30]. Indeed, the small size of NPs conveys distinct physiochemical properties to these particles, such as increased reactivity and ease of transport, which cause them to not only have different interactions with erythrocytes from conventional pharmaceuticals, but also to have a higher risk of undesirable toxicity profiles.

### 2.2. The Contribution of Erythrocyte Aggregation to Blood Viscosity and Hemostasis in Connection with Nanoparticle–Erythrocyte Interactions

Erythrocyte aggregation is largely attributed to the discord morphology and electrostatic interactions between erythrocytes. At low shear rates, erythrocytes tend to form linear face-to-face cell stacks or arrays referred to as rouleaux [14]. Under physiological conditions these linear arrays contribute to the non-Newtonian behavior of blood and can easily be dispersed by normal shear forces. However, when erythrocyte aggregability is abnormally enhanced, through interactions with NPs for example, these aggregates are more difficult to disperse and can potentially occlude small blood vessels and reduce tissue perfusion [31]. Erythrocyte aggregates are known to increase platelet margination, which in small vessels leads to an increase in blood viscosity in the center of the vessel, and a decrease in viscosity near the vascular wall. This is called the Fahraeus effect and can lead to increased endothelial activation and platelet aggregation [17]. 

**Figure 1:**
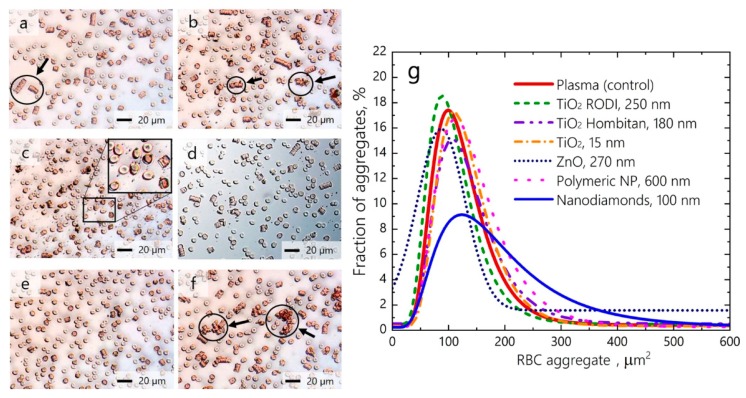
Relative size distribution of erythrocyte aggregates observed by conventional optical microscopy under the influence of (**a**) polymeric NPs 600 nm; (**b**) TiO_2_ NPs 250 nm; (**c**) TiO_2_ NPs 180 nm; (**d**) TiO_2_ NPs 15 nm; (**e**) ZnO NPs 270 nm; and (**f**) nanodiamonds 100 nm. (**g**) Distribution of erythrocyte aggregates by occupied area based on the quantitative assessment of images in (**a**–**f**) (adapted with permission [32]).

Recent studies have shown that cell–NP interactions can cause significant erythrocyte aggregation with damaging consequences, despite the NPs having little hemolytic activity. More and more studies are including erythrocyte aggregation, in addition to cell viability and hemolysis assays, to demonstrate the hemocompatibility of NPs. A recent study by Avsievich et al. showed that erythrocytes formed dissimilar aggregates with irregular shapes and sizes when incubated with different inorganic and polymeric NPs (Figure 1). Among the NPs studied, nanodiamonds caused the greatest increase in the attraction forces between erythrocyte membranes and led to the formation of large, abnormal aggregates. Polymeric NPs on the other hand, did not significantly influence erythrocyte interaction and only standard erythrocyte rouleaux was observed upon erythrocyte incubation with these NPs [32]. 

Two models of aggregation, namely the ‘bridging’ and the ‘depletion’ model, are commonly used to explain the interactions between macromolecules, such as fibrinogen, and erythrocytes. In the bridging model, macromolecules adsorb onto erythrocyte membranes and ensue attractive bridging forces. Once these attractive forces overcome the normal repulsive forces between membrane surfaces, erythrocyte aggregation occurs. This model assumes that macromolecules form ‘cross-bridges’ that facilitate interactions between adjacent erythrocytes [33]. The depletion model is based on the relatively lower concentration of macromolecules near the cell surface compared to the rest of the cell environment that forms a so-called ‘depletion layer’. Once macromolecules adsorb to the cell membrane, the depletion layer is disrupted, and osmotic pressure pushes the cells together [34]. Many researchers argue that these same models can be used to explain NP-induced erythrocyte aggregation, even though much controversy exits about the preferred model. According to Kim et al., the depletion model can successfully explain erythrocyte aggregation caused by Ag-NPs [35]. Han et al., however, preferred the bridging model to explain the interactions between hydroxyapatite NPs and erythrocytes [36]. It could be that the intrinsic NP properties such as composition, size and shape have a significant influence on cell–NP interactions and largely determine the model that is most appropriate to explain NP-induced erythrocyte aggregation. This again stretches the importance of conducting hemocompatibility studies in a case-by-case manner to evaluate and confirm the safety of NPs.

### 2.3. Erythrocyte Deformability as A Mediator of Blood Viscosity and Hemostasis in Connection with Nanoparticle–Erythrocyte Interactions

Deformability refers to the ability of erythrocytes to change their geometry in response to changes in the blood flow and shear rates [37]. This allows erythrocytes to minimize their resistance to flow, enables them to move through the smallest capillary vessels and ensures the adequate perfusion of all tissues [38]. This unique ability of erythrocytes to adapt their shape in response to environmental factors has a direct influence in blood viscosity. A decrease in deformability prevents erythrocytes from obtaining the desired orientation to flow, which increases flow resistance and subsequently blood viscosity [39]. Additionally, decreased erythrocyte deformability is associated with a heightened thrombotic potential as more rigid erythrocytes can easily occlude micro-vessels, attenuate and alter blood flow and facilitate platelet activation [40]. 

Different NPs, all promising candidates in nanomedicine, have been found to interact with erythrocyte membranes in such a way that the cell’s intrinsic function in oxygen transport is threatened and its commended deformability is altered [41]. Hemorheological studies on mesoporous silica NPs, PEGylated gold NPs, and nanosized gold quantum dots (GQD) revealed that attachment of the nanomaterials to the erythrocyte membrane significantly restricted its flexibility and impaired its deformability [42,43,44]. By reducing the deformability of erythrocytes, NPs will not only impair effective blood flow but can also augment the accumulation of erythrocytes in the spleen that can potentially hinder the normal functional activity of this vital organ, as seen in the case of mesoporous silica NPs [42]. 

### 2.4. The Influence of Phosphatidylcholine Exposure on Erythrocyte Membranes in Connection with Nanoparticle–Erythrocyte Interactions

Besides hemorheology, erythrocytes also contribute towards coagulation and blood clotting through exposure of phosphatidylserine (PS) on their membrane surfaces [45]. In normal and quiescent erythrocytes phosphatidylserine, a key lipid of the cellular membrane, is arranged in an asymmetrical fashion. However, when erythrocytes are activated or damaged under conditions of high shear stress, complement attack or oxidative stress, this asymmetrical distribution is disrupted with consequent exposure of PS on the membrane surface [15]. This provides a procoagulant surface for the assembly of coagulation complexes (i.e., intrinsic tenase and prothrombinase) that ultimately leads to thrombus formation. Several research groups have found that erythrocytes, in addition to activated platelets, are the main suppliers of procoagulant surfaces in vivo which highlights their significant role in hemostasis and coagulation [16]. Moreover, the above-mentioned mechanisms of erythrocyte activation or damage can potentially be enhanced by nanomaterials present in the blood. An elegant study done by Ran et al. looked specifically at the influence of Fe_3_O_4_ magnetic NPs on PS expression in erythrocytes to evaluate their nanotoxicity. Their research showed that these extensively explored NPs cause PS externalization on the erythrocyte surface through the induction of oxidative stress. This led to unavoidable eryptosis and subsequent changes in hemorheology, erythrocyte rigidity and aggregation which can alter the thrombotic potential of blood and again highlight the great importance of ensuring NP hemocompatibility [46]. A more recent study evaluated the potential adverse and sensitizing effects of two discrete NPs (polystyrene nanoparticles (PSNP) and lysozyme dextran nanogels (LDNG)) and found that PSPN alone significantly sensitized erythrocytes to oxidative, mechanical and osmotic damage and increased erythrocyte PS exposure [47].

## 3. Platelet Function in Hemostasis and the Mechanisms Involved in Nanoparticle Hemocompatibility

Platelets are well renowned for their pivotal role in preventing blood loss by forming a hemostatic plug following vascular injury. However, the role of platelets in normal hemostasis goes beyond simple clot formation during hemorrhaging and is in fact a continuous physiological process that keeps the vascular system intact [48]. Everyday bruising and the heightened risk of bleeding when platelet numbers are low are evidence enough that vascular damage is a common event which requires daily management by platelets [49]. Yet, the idea that the regulation of hemostasis is the sole function of platelets has long been discarded and they are now also recognized for their distinct role in innate immunity, vascular inflammation, combatting infection, regulation of tumor growth and angiogenesis [50,51]. Platelets are one of the most versatile and critical cells in circulation and interference of NPs with any of their distinct functions can have debilitating effects. During primary hemostasis, platelets go through three critical stages, namely platelet adhesion, activation and aggregation to secure hemostasis. The influence of nanomaterials on all three these stages must be thoroughly evaluated during hemocompatibility testing. 

### 3.1. Platelet Adhesion in Hemostasis and The Effect of Nanoparticle Interactions

Adhesion is a rapid process that takes place within seconds of vascular injury and entails the deceleration of rapidly moving platelets to allow adherence to the vascular wall [52]. Owing to platelet margination by larger molecules, these cells are already in close proximity to the vascular endothelium and complexes can easily form between the platelet-specific receptors, such as glycoprotein (GP) Ib-V-IX, and the cell adhesion ligand von Willebrand factor (VWF), which acts as a bridge between the platelet surface and endothelial collagen [53]. 

**Figure 2:**
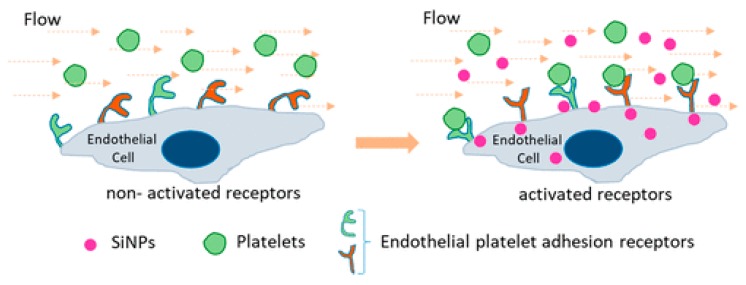
Increased platelet adherence to the surface of endothelial cells in the presence of silica nanoparticles [55].

Silica NPs have been intensely studied for their use in nanomedicine fields including drug delivery systems, bioimaging and diagnosis. However, an increasing amount of studies have found that engineered silica NPs may hold adverse implications for vascular hemostasis that will limit their biomedical application [54]. Saikia et al. investigated the effect of silica NPs on platelet adhesion under flow conditions and found these NPs enhanced platelet–endothelial cell interaction though the increased expression of PECAM-1, a platelet endothelial cell adhesion molecule, on the surface of cells (Figure 2) [55]. In addition, a very recent study found that silica NPs can cause hypercoagulation by inducing endothelial damage with subsequent increased expression of the same adhesion molecule (PECAM-1) as well as tissue factor (TF), a potent coagulant, by endothelial cells [56]. 

### 3.2. Platelet Activation in Hemostasis and The Effect of Nanoparticle Interactions

Platelets can be activated by numerous stimuli from various origins, including the platelets themselves that secrete activating agonists like adenosine diphosphate (ADP) and thromboxane A2, after adhesion to the endothelium. Other common endogenous stimuli include collagen, thrombin, epinephrine and complement C5b-9 [57]. Activation results in conformational changes (platelets become spherical with prominent pseudopodia) that lead to increased ligand binding and secretion of procoagulant factors from their granular contents. Platelets contain three types of storage granules (alpha, dense and lysosome granules) that harbor a wide range of substances with critical functions, not only in hemostasis, but also in inflammation and vascular repair (Table 1). Inhibited release of these substances can be both congenital or acquired (e.g., drug/NP induced) and can significantly impair platelet function [53]. For example, during extensive hemocompatibility testing, Krajewski et al. found Ag-NPs caused platelet activation by increasing the secretion of agonistic substances from platelet alpha granules [25]. The wide spectrum of substances released from platelet granules showcases just how versatile and significant platelet function is. 

### 3.3. Platelet Aggregation in Hemostasis and the Effect of Nanoparticle Interactions

Once platelets become activated, conformational changes allow the integrin GPIIb/IIIa to bind to various ligands (e.g., fibrinogen, thromboxane A2) and catch passing platelets to build the platelet plug further. Interaction between this glycoprotein receptor and circulating proteins is essential to the final stage of platelet aggregation and helps stabilize the formed platelet aggregate [58]. Inhibitors of platelet aggregation, such as prostacyclin, balance the aggregating effects of activating factors, such as thromboxane A2, and prevent excessive thrombus formation to ensure the patency of the vascular lumen [53]. 

One of the key determinants of NP interactions with platelets is the surface chemistry of the particles. Ionizable sialic groups on the cellular membrane of platelets confer a net negative charge to the platelet surface which can be neutralized by positive charges on the surface of NPs through cross-bridge formation. Cationic NPs can therefore facilitate platelet–platelet interactions and enhance platelet aggregation [59]. Indeed, cationic polypeptides have been found to raise platelet aggregation by forming bridges between platelets and reducing their surface charge [1]. 

**Figure 3:**
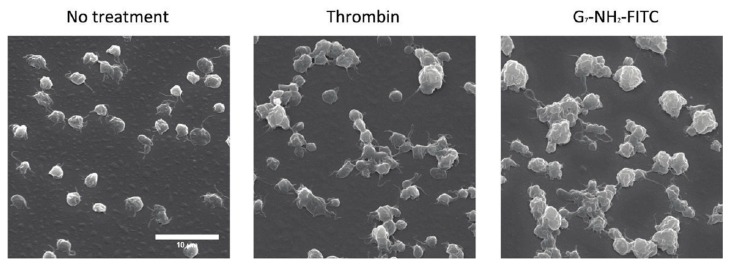
SEM images of platelets, taken at 2500× magnification, after treatment with saline, thrombin (0.1 U/mL) or G7-NH_2_ dendrimer (100 μg/mL). Notice the disheveled membrane morphology as well as increased number and size of platelet aggregates after treatment of platelets with G7-NH_2_ dendrimer (right hand panel) [63].

However, interaction through electrostatic surface charges is not the only mechanism whereby NPs lead to platelet aggregation, and each nanosystem must be investigated as an independent case [60]. Amongst the different nanosystems, dendrimers stand out in terms of their ability to induce platelet aggregation; numerous types of dendrimers, including polyamiodamine (PAMAM), polypropyleneimine (PPI), triazine and carbosilane dendrimers, have been found to cause aggressive thrombus formation [61]. PAMAM dendrimers specifically have been extensively studied for their hemotoxicity and effects on blood platelets, especially after Greish et al. found amine-terminated PAMAM dendrimers to cause a lethal, disseminated intravascular coagulation-like condition in CD1 mice [62]. Jones et al. demonstrated that amine-terminated PAMAM dendrimers bind to and alter the morphology of platelets and lead to an increased number and size of platelet aggregates, even when compared to thrombin activated platelets (Figure 3) [63].

### 3.4. In Vivo Platelet Toxicities Induced by Nanoparticles

**Figure 4:**
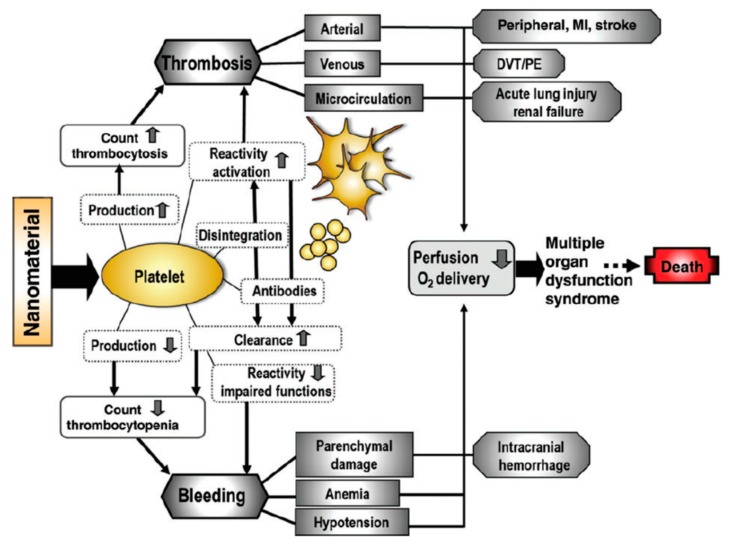
The potential in vivo adverse effects resulting from nanomaterial interactions with blood platelets. Abbreviations: DVT, deep venous thrombosis; MI, myocardial infarction; PE, pulmonary embolism [65].

However, the potential in vivo toxicities that can arise from NP interactions with platelets are perhaps the most significant encouragement to carefully evaluate any possible interactions of engineered NPs with blood platelets [64]. These possible in vivo effects and clinical expressions are summarized in Figure 4. The exact mechanisms and eventual clinical effects of NP–platelet interactions are extremely diverse and case-specific due to the substantial influence of particle composition, size, shape and surface chemistry on these interactions [1]. It must also be kept in mind that nanomaterials, with diagnostic or therapeutic applications, can potentially be administered to patients who are already severely ill. These patients might show disproportionate hemostasis, inflammatory responses and dysfunctional organs that can potentially be tipped towards perilous clinical manifestations, by even the smallest interaction of NPs with platelets [65].

## 4. Leukocyte Function in Hemostasis and the Mechanisms Involved in Nanoparticle Hemocompatibility

Another cellular constituent of blood, which is often overlooked in terms of contribution to hemostasis, is represented by leukocytes. These cells are subdivided into monocytes, granulocytes and lymphocytes (Table 2) and play a critical role in inflammation, immunity and host defense systems [66]. However, recent studies have also found that these cells actively contribute to normal hemostasis as well as thrombotic conditions [67]. In fact, numerous authors now argue that the coagulation and innate immune systems are inseparable due to common mediators and activating factors that intricately link these two systems [68]. 

Activated platelets regulate the proinflammatory activity of leukocytes through two mechanisms, namely degranulation, which exposes leukocytes to activating factors such as chemokines, and P-selectin exposure, which is a surface receptor that interacts with the leukocyte surface receptor PSGL-1 [72]. This interaction not only recruits leukocytes to the cite of vascular injury, but also induces leukocytes to express considerable quantities of tissue factor (TF), a critical propagator of the coagulation cascade [73]. Another significant effect of activated platelets on leukocytes is the formation of neutrophil extracellular traps (NET) which, in addition to their well-established role in antimicrobial defense, have now been found to also have thrombogenic properties with implications in stroke, myocardial infarction (MI) and DVT [74]. This is clear evidence that, although platelets usually regulate inflammation through leukocyte activation and recruitment, leukocytes can also regulate blood coagulation. 

Indeed, quiescent leukocytes play a vital role in maintaining blood fluidity by expressing the anticoagulant factors, endothelial protein C receptor (EPCR), thrombomodulin (TM) and tissue factor pathway inhibitor (TFPI), under normal physiological conditions [75]. However, under proinflammatory or apoptotic conditions, leukocytes can rapidly assume a procoagulant role by releasing proinflammatory and procoagulant mediators such as granular enzymes, cytokines and damage-associated molecular patterns (DAMPs). In addition, the surface of leukocytes, just like that of erythrocytes and platelets, can provide a surface for coagulating factors to accumulate and a thrombus to form [68]. 

**Figure 5:**
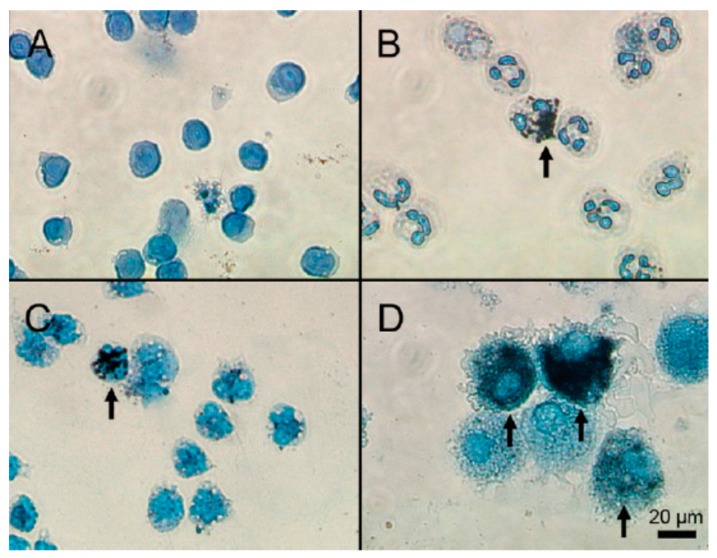
Cytospin preparations of (**A**) lymphocytes; (**B**) neutrophil granulocytes; (**C**) monocytes; and (**D**) macrophages after incubation with CTAB-coated AuNPs. Seedless deposition results in black spots or areas that indicate the presence of gold nanoparticles (indicated with arrows) [78].

This strong interrelation between the immune and coagulation systems makes leukocyte activation an important parameter during the hemocompatibility evaluation of nanomaterials, in addition to its normal inclusion in immunotoxicity studies [76]. Furthermore, engineered NPs in the blood are often recognized as foreign bodies and phagocytosed by circulating leukocytes. This not only brings NPs into unswerving contact with leukocytes but can also lead to the significant accumulation of NPs within these cells [77]. Figure 5 shows the uptake of cetyltrimethylammonium bromide (CTAB)-stabilized gold NPs by lymphocytes, granulocytes, monocytes and macrophage. NPs were internalized rapidly (within 15 minutes) and in large amounts by macrophages and by a lesser extent also by granulocytes and monocytes [78]. 

One reason for the high accumulation of NPs within leukocytes is plasma proteins that adsorb to the surface of NPs, marking them for clearance by leukocytes [79]. This adsorption is in effect the base of all hemocompatibility, as the adsorbed plasma proteins effectively coat the NPs and prevent the direct contact of blood cells with the material itself. This is often referred to as the NP’s ‘true identity’ as determined by its encountered biological environment, compared to its ‘synthetic identity’ that is determined by the intrinsic properties of the material [80]. The protein adsorption process is a dynamic one with abundant, low-affinity proteins rapidly absorbing to the particle surface and later being replaced by higher affinity but lower abundance proteins as time progresses. This is called the Vroman effect (first described by Leo Vroman) and stretches the importance of studying the kinetics and association/dissociation constants of proteins as well as conducting hemocompatibility tests over different and extended periods of time [81,82]. 

The proteins that preferentially bind to polymeric NPs, liposomes, carbon nanotubes and metal oxide particles are albumin, fibrinogen, apolipoprotein, complement and immunoglobulins [83]. Each of these proteins has distinct effects on the in vivo fate of the NPs it adheres to. Human serum albumin is the most abundant plasma protein and rapidly adsorbs to NPs, potentially masking them from the mononuclear phagocytic system (MPS) and reducing their cellular uptake [83]. Apolipoprotein, immunoglobulins and complement components, on the other hand, cause the opsonization of NPs that makes them more visible to phagocytic cells and triggers pro-inflammatory responses towards the NPs [84]. Recently, inflammatory responses have also been found towards fibrinogen-adsorbed NPs that even showed increased thrombogenicity and platelet adherence to the particle surface [85]. Furthermore, the adsorption of plasma components can modify the size, aggregation state and interfacial composition of nanomaterials and should therefore be carefully considered when evaluating the interactions and toxicities of NPs within the blood.

**Figure 6:**
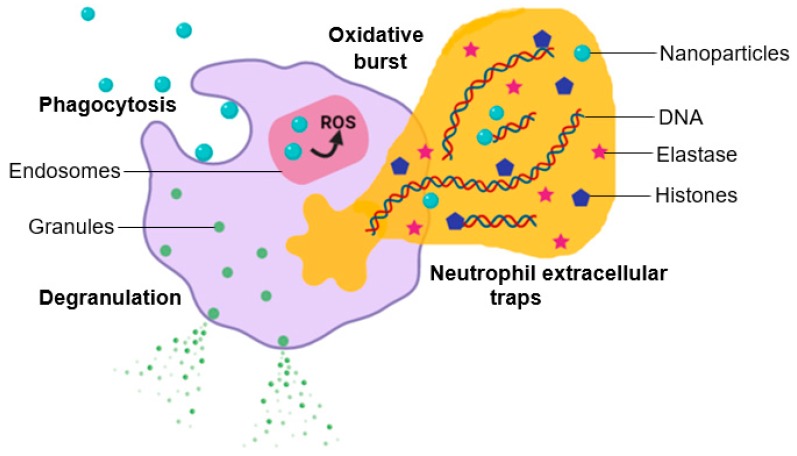
Potential effect of nanoparticle–neutrophil interactions.

To conduct a comprehensive study of the potential adverse effects of NPs on leukocytes, their influence in the characteristic functions of leukocytes must be considered. These include chemotaxis, cytokine secretion, oxidative burst and phagocytosis, which should be studied in conjunction with basic changes in leukocyte morphology and apoptosis caused by NPs [86]. Figure 6 provides a schematic depiction of certain effects that NPs can have on leukocyte function including oxidative burst, degranulation and NET formation.

### 4.1. The Effect of Nanoparticle–Leukocyte Interactions on Chemotaxis

Chemotaxis entails the recruitment or attraction of leukocytes to an environment with a higher concentration of toxic, sensory chemicals thereby allowing them to move towards intruding substances and phagocytose them [84]. Kojouri et al. set out to determine the influence of nano-selenium and sodium selenite on the chemotactic activities of neutrophils in sheep [87]. They found that selenium NPs caused a greater and longer-standing increase in the chemotactic activity of neutrophils compared to sodium selenite. Durocher et al. found that both AuNP(+) and AuNP(–) significantly increased the chemotactic activities of human neutrophils and led to a prominent induction of leukocyte infiltration in a murine air pouch model, whereby they concluded that these NPs have both in vitro and in vivo pro-inflammatory properties [88]. In contrast, Herrmann et al. found no net-movement of neutrophils towards carbon-coated metal nanomagnets incubated in blood plasma and concluded that these NPs do not induce the release of chemotactic mediators [89].

### 4.2. The Effect of Nanoparticle–Leukocyte Interactions on Cytokine Secretion

One method whereby leukocytes react to foreign irritants is degranulation and the subsequent release of digesting enzymes (i.e., proteinases such as elastase) and other factors (i.e., cytokines such as interleukins and monocyte chemotactic proteins) that aid the removal of irritants. Recent studies also show that certain cytokines released by neutrophils have a distinct role in the resolution of the inflammatory process [90]. NPs that interfere with this critical part of leukocytes’ role in infection, inflammation and tissue repair can have deleterious effects on human health ranging from severe, recurrent infections to excessive inflammatory conditions [91]. 

Over the years, numerous studies have proved the stimulating effect of chitosan NPs on leukocyte secretion of different cytokines including IL-18, IL-1β and granulocyte-colony stimulating factor (G-CSF) which Nolte et al. found to be upregulated by more than 40% [92,93,94,95]. A very recent study, done on chitosan-based NPs and oligosaccharides in cyclosporin treated mice, found the NPs to be superior to chitosan itself in terms of their immunomodulatory abilities, which was attributed to the unique characteristics and biological activities of the NPs. The authors argue that physiochemical properties of NPs, per se, influence their interaction with immune cells to induce both desirable effects and unavoidable, undesirable nanotoxicity [96].

A common technique to determine leukocyte activation and degranulation is by measuring the release of elastase from neutrophils. Fang et al. compared the influence of cationic lipid and polymeric (PLGA) NPs on leukocyte elastase release and found both to induce a threefold increase in elastase, even though the lipid NPs generally had a greater effect on the neutrophils [97]. The results showed that the composition of the NP core as well as the type of surfactants used on the surface have a determining effect on the neutrophil response. Such induced elastase release, caused by NPs, is troublesome since elastase secreted by neutrophils has the ability to destroy the extracellular matrix of cells and increased activity thereof can potentially lead to tissue and organ injury [98]. 

Another interesting study looked at the direct effects of drug-free poly(ε-caprolactone) lipid-core nanocapsules (LNCs) on leukocytes and found that these particles inhibit the secretory abilities of leukocytes in both basal and stimulated conditions. This suggest that even carrier particles can have distinct effects on leukocyte function and must be carefully evaluated and then used to guide their therapeutic applications to avoid discrepancies between the immunological effects of the carrier and the drug itself [99].

### 4.3. The Effect of Nanoparticle–Leukocyte Interactions on Oxidative Burst

In combination with cytokine release, oxidative burst is another method employed by leukocytes to eliminate unwanted or exogenic substances like microbes and NPs [85]. Reactive oxygen species (ROS) are natural by-products of oxygen metabolism in neutrophils and include superoxide anion (O_2_^•−^) and hydrogen peroxide (H_2_O_2_). These substances can react with hypochlorous acid within the cell to produce free radical species that help in the degradation of NPs and other exogenic substances. However, stressful conditions such as infection, inflammation or exposure to engineered or environmental NPs may significantly increase ROS production in leukocytes that may disturb homeostasis and cause cellular damage and tissue injury [100].

A recent study showed that quantum dots thrust neutrophils into a hyperactive state with an overproduction of ROS and a more pronounced respiratory burst compared to the control [101]. Another study investigated the influence of C60 fullerene NPs and their nanocomplexes with anticancer drugs on intracellular ROS generation by leukocytes. The results showed a sharp increase in ROS production after treatment with all the studied NP preparations and an eightfold increase caused by C60 fullerene NPs alone. It was therefore concluded that these particles are strong inducers of intracellular ROS generation and can possibly counteract the pro-oxidant effects of anticancer drugs like cisplatin [102].

A fascinating study done by Pujari-Palmer et al. evaluated the influence of NP morphology on the acute inflammatory response and oxidative burst. The fiber morphology caused the highest ROS production in both granulocytes and monocytes with 45% and 40% increases, respectively. This excessive free radical production proved toxic to both the phagocyte and the surrounding cells, with blebbing occurring within 1 hour of contact with the fibers. It was concluded that fibers, when compared to sheets and dots, cause the highest acute inflammation and that NP morphology, independent of charge and chemistry, has an important influence on NP–cell interactions and the subsequent induced inflammatory response [103].

**Figure 7:**
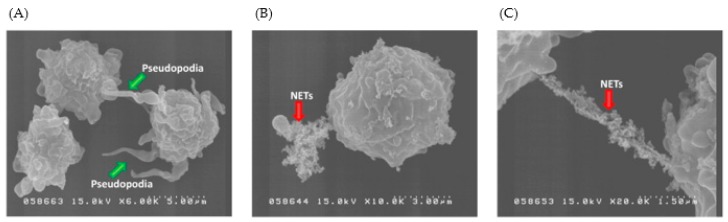
The morphology of human neutrophils examined by SEM after treatment of (**A**) cationic SLNs (55 lg/mL) at a magnification of 6000×; (**B**) cationic SLNs (55 lg/mL) at a magnification of 10,000×; and (**C**) cationic SLNs (55 lg/mL) at a magnification of 20,000× [107].

Another important observation is that a positive charge on the surface of NPs can, in addition to warranting effective cellular drug delivery, also ensue immunotoxicity through the stimulation of inflammatory immune responses. Several researchers have found that cationic liposomes can stimulate neutrophils and induce oxidative bursts in these cells [10,11,12,13,14,15,16,17,18,19,20,21,22,23,24,25,26,27,28,29,30,31,32,33,34,35,36,37,38,39,40,41,42,43,44,45,46,47,48,49,50,51,52,53,54,55,56,57,58,59,60,61,62,63,64,65,66,67,68,69,70,71,72,73,74,75,76,77,78,79,80,81,82,83,84,85,86,87,88,89,90,91,92,93,94,95,96,97,98,99,100,101,102,103,104,105,106]. Interestingly, there seems to also be a correlation between the increased ROS production caused by cationic liposomes and NET generation in the stimulated neutrophils. Hwang et al. proved that ROS production, as a consequence of cationic solid lipid nanoparticle (SLN) exposure, induced neutrophil migration and pseudopodia extension that ultimately resulted in NET generation (Figure 7) [107]. Similarly, a study done on different liposomes, found those treated with a cationic additive (cetyltrimethylammonium bromide) greatly increased superoxide anion levels that also resulted in NET release from the neutrophils [97]. Recently, Lotosh et al. showed that cationic liposomes containing stearyl amine (SA liposomes) could induce both the production of ROS and NET formation. Their data proved the correlation between these two processes as NET formation could not occur when oxidative bursts of neutrophils were suppressed [108].

### 4.4. The Effect of Nanoparticle–Leukocyte Interactions on Phagocytosis

Before either cytokine release or oxidative burst can occur, interaction between NPs and leukocytes, with subsequent activation of the immune cells, is necessary. This takes place through the initial phagocytic uptake of NPs by leukocytes, which is one of the greatest barriers to effective, targeted delivery of NPs in vivo [109]. Phagocytosis of NPs by leukocytes is such a pertinent event that researchers are now even looking at ways to utilize this occurrence as a target for drug delivery [110]. In addition to being phagocyted themselves, NPs can interfere with the phagocytic abilities of leukocytes, which holds important implications for this critical step in the innate immune defense. 

In a recent study, Ho et al. looked at the number of bacteria ingested by polymorphonuclear (PMN) cells after administration of sub-lethal doses of citrated Ag-NPs to determine the influence of these NPs on the phagocytic abilities of PMN cells. They found that Ag-NPs can significantly suppress the phagocytic ability of PMN cells and suggest that agglomeration of NPs in intracytoplasmic vacuoles restricted the expansion capability of PMN cells which might explain the suppressive phagocytic effects of Ag-NPs [111]. However, NPs can suppress phagocytosis by interfering with any of the three steps involved in phagocytosis, namely attachment of the target particle, extension of pseudopodia around the attached particle and engulfment of the attached particle into a phagosome. If NPs cause defeat in any one of these steps, the result will be impaired phagocytosis with a definite alteration in the immune and inflammatory responses of neutrophils [112]. 

On the other hand, Babin et al. found that several NPs can, in addition to being taken up by phagocytosis themselves, directly enhance the ability of neutrophils to phagocytose other particles, including opsonized erythrocytes and fluorescent latex beads. The results revealed that NPs can act in the same way as granulocyte macrophage colony-stimulating factor (GM-CSF) to enhance phagocytosis by a Syk-dependent mechanism. According to the authors of this study, the influence of naked NPs on the biology and functioning of neutrophils (especially whether NPs can alter the phagocytic process themselves) has been greatly neglected in the past and must be carefully evaluated during future studies involving NP interactions with immune cells [113]. 

### 4.5. Cytotoxicity as a Result of Nanoparticle–Leukocyte Interactions

In addition to the above-mentioned studies where the effect of NPs on leukocyte functions are scrutinized, basic cytotoxic effects such as induced apoptosis, necrosis and morphological changes in the immune cells must not be overlooked. Different mechanisms have been proposed to explain the cytotoxic effect of NPs on leukocytes. These include cellular membrane damage as indicated by lactate dehydrogenase (LDH) release, imbalances between oxidant and anti-oxidant processes that can lead to ROS generation and DNA damage as well as disruption of the autophagy and endo-lysosomal pathways [77,114]. Colognato et al. demonstrated that cobalt (Co) NPs are rapidly internalized by human leukocytes where they cause significant DNA strand breakage [115]. Jiang et al. also investigated the cellular toxicity of Co-NPs towards leukocytes and found that both Co-NPs and released Co^2+^ ions damaged the cellular membrane in a concentration- and time-dependent manner that corresponded with the cell viability assay results. They also demonstrated a statistically significant, concentration-dependent increase in DNA damage induced by Co-NPs at both 3 and 6 μM. Lastly, they found that imbalance of the oxidative/antioxidative system may play an important role as these NPs markedly interfered with the activity of several anti-oxidant enzymes in the lysosomes [116]. 

Autophagy and lysosomal dysfunction are considered underlying mechanisms of NP cytotoxicity. NP endocytosis typically results in concentration of NPs inside lysosomes, making the lysosomal compartment the most common intracellular site of NP sequestration and degradation [117]. Dysfunction of the endo-lysosomal pathway have been associated with several NPs, including carbon nanotubes, PAMAM dendrimers and polystyrene nanospheres [118,119,120]. Lysosome membrane permeabilization (LMP) is a common form of lysosomal dysfunction that can have detrimental outcomes including ROS generation, apoptosis, cytosolic acidification and necrosis [121]. Autophagy is generally considered a nonselective response to cellular stress but is also a method whereby lysosomes remove intracellular pathogens, damaged organelles and long-lived proteins. Autophagic dysfunction involves either excessive induction of autophagy or blockade of autophagy flux and is recognized as a mechanism of cell death that results in apoptosis or autophagic cell death (also referred to as type II programmed cell death) [122]. Nanoscale neodymium oxide is a potent inducer of autophagy through an oxidative stress mechanism, while fullerene and Au-NPs are known to block the autophagy flux resulting in autophagosome accumulation and cell death [123,124]. 

## 5. The Influence of Surface Chemistry on Nanoparticle Hemocompatibility

In terms of NP hemocompatibility and NP–blood cell interactions, NP surface chemistry plays an irrefutable role [28]. Not only does the surface chemistry determine the protein corona composition but it also conveys critical properties to NPs that will influence their cellular uptake and potential toxicity mechanisms. The most dominant surface properties that govern the hemocompatibility of NPs are surface charge, geometry, porosity and surface functionalization with specific polymers or functional groups [125].

### 5.1. Surface Charge as A Determining Factor of Nanoparticle Hemocompatability

It is widely accepted that cationic NPs will favorably interact with the negatively charged cell membrane surface, allowing these NPs to easily translocate across the cell membrane with subsequent high levels of cellular internalization [126]. Anionic NPs on the other hand are thought to have unfavorable interactions with the cellular membrane due to repulsive forces between the two negatively charged surfaces and therefore show poor cellular internalization [62]. This makes cationic NPs the preferred vehicle for drug and gene delivery. Unfortunately, numerous studies have found different positively charged NPs to have hemotoxic effects that supersede that of similar anionic NPs [127].

Like most cell membranes, the erythrocyte surface is negatively charged and can readily interact with cationic NPs. Indeed, Han et al. found that HAP-NPs exhibited surface charge dependent erythrocyte aggregation due to electrostatic interactions between NPs and the erythrocyte membrane which resulted in unstructured agglutination. Surface functionalization of the HAP-NPs with negatively charged groups annulated the erythrocyte aggregating effects of these NPs [36]. Platelets can also be negatively affected by cationic NPs, as in the case of cationic PAMAM dendrimers that significantly altered platelet morphology and activation state through the release of α-granule contents after interaction with the platelet membrane and internal cytoskeletal structures. The same study showed that neutral and anionic PAMAM dendrimers have no effect on platelet function and morphology, confirming the effect of cationic PAMAM dendrimers to be surface-charge-dependent [63]. Positively and negatively charged Au-NPs was found to have distinctly different effects on leukocyte activation, function and cell viability. Cationic Au-NPs activated leukocytes to a greater extent than anionic Au-NPs even though both positively and negatively charged Au-NPs increased the chemotactic activity if these cells. Anionic Au-NPs on the other hand caused significant leukocyte apoptosis while cationic Au-NPs did not [88]. This coincides with another study that found both negatively charged Au-NP_20_ and negatively charged Au-NP_70_ to be proapoptotic towards leukocytes [128]. Surface charge can clearly alter the toxicological profile of NPs towards blood cells and should be considered when investigating the hemocompatibility of different NPs.

### 5.2. Geometry as a Determining Factor of Nanoparticle Hemocompatability

The importance of NP geometry or shape is often overlooked in terms of nanotoxicity but can easily be exploited to improve the hemocompatibility of NPs. NP geometry determines the effective surface area of the NP, the tendency of NPs to agglomerate, the interactions of NPs with plasma proteins, the mechanism and extend of cellular uptake and therefore also the potential toxicity of NPs [129].

However, the importance of NP geometry in terms of hemocompatibility is clearly seen in the case of amorphous silica NPs where the extent of hemolysis is concentration- and geometry-dependent. With increasing concentration, the impact of NP geometry becomes more pronounced and geometries with a low aspect ratio, such as spherical NPs, induce significant hemolysis while geometries with a high aspect ratio is associated with negligible hemolytic activity [29]. The geometry of the NP directly influences its external surface area and curvature which in turn influences its hemolytic activity by altering the magnitude of the NPs’ binding energy to the erythrocyte membrane. In the case of spherical NPs, their large external surface area and small curvature renders the hemolytic process thermodynamically favorable, thereby increasing their hemolytic activity [42].

Another critical effect of NP geometry is its influence on particle margination. As noted earlier, NP- and platelet margination is central to normal hemostasis, NP hemocompatibility and the therapeutic efficacy of NPs. NPs with complex geometries and high aspect ratios demonstrate greater margination, bringing them into close contact with the endothelial wall and platelet-dense region of capillaries [21]. Decuzzi et al. effectively demonstrated the geometry-dependent margination of silica NPs where discoidal NPs exhibited a greater margination tendency compared to quasi-hemispherical and spherical NPs [130]. This heightened margination can have a significant influence on the degree of NP–platelet interaction and potentially increase the risk of thrombus formation [17].

NP geometry also defines the cellular uptake efficacy, with rod-shaped NPs internalized most readily, followed by spheres and cylinders, while cubes are not easily internalized [131]. Bartneck et al. hypothesized that the morphological similarity of nanorods to the protein capsules of viruses, which are internalized by leukocytes with striking efficacy, supports the greater uptake of NPs with this geometric shape. The degree of NP cellular uptake directly influences the cytotoxicity of the NP and must therefore be carefully considered during NP synthesis and design [78]. Furthermore, Pujari-Palmer et al. found that NP geometry can have a pertinent influence on leukocyte functioning where fiber-shaped HAP-NPs inducing the greatest amount of ROS generation, caspase 3/7 activation and acute in vivo inflammation compared to other geometries such as rods, dots and sheets [102].

### 5.3. Porosity as a Determining Factor of Nanoparticle Hemocompatability

According to Park et al., NP porosity and crystallinity is a pertinent driving force behind the hemotoxic effects of certain NPs and must be carefully considered when designing new nanosized biomaterials [132]. The difference in hemotoxicity of NPs with different porosities is ascribed to the interaction of plasma proteins with NP pores. Larger pores allow proteins to adopt different conformations that will expose different binding sites, leading to different interactions with blood cells [133]. According to Greish et al., the biological environment perceives porous NPs as completely different from solid NPs since the pores influences the type of plasma proteins that adsorb to the surface as well as their molecular arrangements [62]. Ferraz et al. confirmed the definitive effect of porosity on NP hemocompatibility by showing that even small changes in the nanopores of alumina NPs significantly alter their hemocompatibility [134].

The hemotoxicity of silica NPs is undeniable, with adverse effects witnessed towards erythrocytes, platelets and leukocytes. Yu et al. studied the impact or NP porosity on human erythrocytes and found that nonporous Stöber NPs caused an immediate onset of hemolysis while no hemolytic activity was observed for mesoporous silica NPs [135]. On the other hand, amorphous silica NPs were found to induce major platelet aggregation through different mechanisms including the upregulation of selectin P expression and NO release from the platelet surface [54]. Additionally, both nonporous and mesoporous silica NPs caused acute toxicity in human leukocyte cells by damaging to the plasma membrane and increasing ROS generation [136]. These studies illustrate that surface porosity can have conflicting effects on the compatibility of NPs with different blood cells. This is in agreement with the results of Ferraz et al., that showed NPs with small pores (20 nm) to be 100% more thrombogenic than similar NPs with larger pore sizes (200 nm), while the NPs with larger pore sizes significantly activated platelets and small pore size NPs did not [134].

### 5.4. Surface Modification as A Determining Factor of Nanoparticle Hemocompatability

One of the main strategies to improve the hemocompatibility of NPs is surface modification which alters chemistry and properties of the NP surface that comes into direct contact with blood. This can often mask the inherent toxicity of the NPs, extend their circulation half-life and drastically alter their interactions with blood cells [101]. Herrmann et al. covalently attached polyethylene glycol (PEG) chains to iron oxide NPs and studied the interaction of these surface functionalized NPs with erythrocytes, platelets and leukocytes to determine if PEGylation improved NP hemocompatibility, as is widely accepted. Their results showed negligible hemolysis upon exposure to PEGylated iron oxide NPs and confirmed the absence of erythrocyte membrane damage. The PEGylated iron oxide NPs also had no effect on platelet aggregation or function, suggesting that these functionalized NPs did not interfere with the cellular coagulation system. In fact, incubation with surface functionalized iron oxide NPs resulted in clotting times within the normal range (300–1000 s) while exposure to unfunctionalized iron oxide NPs resulted in more rapid clot formation. Chemotaxis experiments showed that the surface functionalized NPs did not interfere with leukocyte functions nor did they induce apoptosis or necrosis of these cells [89]. This study and numerous others illustrate improved hemocompatibility of NPs after surface functionalization with PEG [26,137,138].

However, an intriguing study done by He et al. found an unexpected negative effect of PEGylated Au-NPs on erythrocyte function. Although these NPs did not induce hemolysis, they did alter erythrocyte deformability and oxygen delivering ability through interaction with the erythrocyte membrane [44]. The authors also found that PEGylation could accelerate the clearance of erythrocytes due to altered functionality, mechanical properties and integrity of erythrocytes which ultimately leads to destruction and premature elimination of these cells. Hakim et al. also studied PEGylated Au-NPs and found that these NPs could survive in the blood for up to one month. The authors speculate that this will result in a long-lasting decrease in the oxygen delivering ability of erythrocytes, that may lead to clinical manifestations such as organ dysfunction and hypoxia [139], demonstrating that NP surface modifications must be made with extreme care.

Kim et al. studied the influence of three different surface functionalized (non-functionalized, hydroxylated and carboxylated) graphene quantum dots (GQDs) on the hemorheological properties of erythrocytes. They found that carboxylated GQDs had more substantial hemolytic activity and caused more significant changes in erythrocyte deformability and aggregation than non-functionalized or hydroxylated GQDs [43]. No clarity was given as to why carboxylated GQDs were more toxic that non-functionalized or hydroxylated GQDs, but Guo et al. has reported an increased release of lactase dehydrogenase (LDH) and ROS generation with carboxyl graphene that could possibly explain the greater toxicity of carboxylated GQDs [140].

From the above-mentioned studies it is apparent that surface chemistry plays an important role NP–blood cell interactions and can have a significant influence on the overall hemocompatibility of NPs. However, it must be considered that even though certain trends, such as greater toxicity of cationic NPs compared to anionic NPs, can be identified in the discussed studies, this should by no means be inferred upon all NPs. Each newly engineered NP must be reviewed as an independent, solitary case that has the potential to interact with blood cells in a previously unseen manner.

## 6. Conclusions and Future Perspectives

The blood is a unique biological fluid with every component playing a distinct role in the effective functioning of the intricate system. This biological fluid is responsible for the transport of all NPs to their target tissues, and it is important that researchers confirm the compatibility of engineered NPs with all three cellular constituents of blood, namely erythrocytes, platelets and leukocytes. This will not only give a more accurate and complete picture of the engineered NP’s hemocompatibility but will also help ensure its optimum safety and enhance its effective translation into clinical application.

Erythrocytes mainly contribute to hemostasis through hemorheological mechanisms such as pseudoplastic flow and axial margination. When NPs interact with erythrocytes they can induce hemolysis, increase erythrocyte aggregation or decrease erythrocyte deformability that will prevent erythrocytes from performing their basic functions in hemostasis. NPs can also induce phosphatidylserine exposure on erythrocyte membranes that will create a pro-thrombotic surface for platelets to adhere to and a subsequent increased risk of thrombosis [15]. Hemolysis assays are often used to evaluate the hemocompatibility of NPs but cannot be used in isolation to demonstrate the compatibility of NPs with erythrocytes [25]. In the future, researchers can take additional, uncomplicated measures, such as optical analysis and deformability calculations to draw a more realistic and complete picture of the influence of NPs on erythrocytes.

Platelets are well-known for their critical role in hemostasis through hemostatic plug formation. Platelets, however, are also responsible for the overall regulation of hemostasis and play a vital role in immunological and inflammatory responses [50]. NPs can hinder normal platelet functioning by either increasing or decreasing platelet adhesion, activation and aggregation that can result in detrimental clinical manifestations such as intracranial hemorrhaging, MI or stroke [64]. Researchers must therefore take extreme care to ensure that NP–platelet interactions do not deter normal platelet functions in primary hemostasis before the engineered NPs advance to clinical trials.

Leukocytes also play a distinct role in hemostasis through the secretion of procoagulant and anticoagulant factors that link the coagulation and immune systems in an inseparable fashion [73]. Leukocytes are at great risk of NP toxicity since they are the cells responsible for taking up and removing foreign particles from the blood, which often results in the high accumulation of NPs inside immune cells. NPs can interfere with the basic functions of leukocytes such a chemotaxis, cytokine secretion, oxidative burst and phagocytosis in addition to causing more detrimental effects such morphological changes, DNA damage and apoptosis [74]. Cytotoxicity studies as well as evaluation of the basic leukocyte functions should be included in thorough NP hemocompatibility evaluations.

The surface chemistry of NPs has a pertinent influence on their hemocompatibility and interactions with blood cells. Surface charge, geometry, porosity and surface functionalization with specific polymers or functional groups are the most important surface properties of NPs in terms of their hemocompatibility and must be carefully considered during the assessment of NP hemocompatibility [125].

Within the scope of research and development, it is critical to view each newly engineered NP as an individual case without conferring any characteristics of similar particles or the bulk material to the engineered NP. Even slight changes to an existing and well-studied particle can result in an entirely new toxicological profile that must be evaluated from scratch [5]. Shvedova et al. fittingly conferred a quote by Norman Augustine: “one should expect that the expected can be prevented, but the unexpected should have been expected” to NPs, describing nanotechnology as an ever-moving target that can be expected to induce toxicological outcomes in unanticipated ways [141]. This stretches the importance of evaluating NP hemocompatibility from every available cellular angle, while expecting to continually uncover new and advanced mechanisms of NP hemotoxicity in the years to come.

Yet the value of understanding NP–blood cell interactions goes beyond improving NP hemocompatibility. This knowledge is central to new and upcoming research on synthetic blood components, artificial blood production and modified blood cell components for novel oxygen delivery to tissues affected by ischemia and trauma [142]. All these new technologies involve nano-bioengineering and NP-based products for which a clear understanding of NP–blood interactions is imperative. Furthermore, the investigation of NP–blood interactions could open the possibility of understanding blood-borne infections or illnesses caused by bacteria and viruses [143].

## Figures and Tables

**Table 1:** Types of platelet storage granules and factors they release (adapted with permission [51]).

Granules	Content Class	Factors Released
Alpha granules	Adhesive glycoproteins	VWF, thrombospondin, P-selectin, fibrinogen, fibronectin, vitronectin
	Coagulation factors	Plasminogen, kininogens, protein S, factor V, factor XI, factor XIII
	Growth factors	IGF, EGF, PDGF, TGF-β
	Angiogenic factors	PF4 inhibitor, VEGF
	Protease inhibitors	C1-inhibitor, PAI-1, TFPI, α2-antiplasmin, α2-antitripsin, α2-macroglobulin
	Immunoglobulins–chemokines	IL8, IL1β, CD40, CXCL4 (platelet basic protein/NAP-2), CXCL (PF4), CXCL1, CXCL5, CCL5 (RANTES), CCL (MIP-1α)
	Proteases	MMP2, MMP9
Dense granules	Amines	Serotonin, histamine
	Bivalent cations	Ca^2+^, Mg^2+^
	Polyphosphates	ADP, ATP, GDP, GTP
Lysosome granules	Enzymes	Acid proteases, glycohydrolases
Other soluble mediators	NO, thromboxane A2, defensins, PAF	

**Table 2:** Cellular players of innate immunity, their sub-types and functions.

Leukocyte	Sub-Type	Function	Reference
1. Granulocytes/polymorphic nuclear cells (PMNs)	Neutrophils	Scavenger of invading pathogens	[69]
	Basophils	Release of inflammatory and anticoagulant mediators	
	Eosinophils	Production of angiogenic and profibrogenic factors	
2. Monocytes/mononuclear cells	Monocytes/macrophage	Phagocytosis	[70]
3. Lymphocytes	T cells	Orchestration of adaptive immunity	[71]
	B cells	Humoral immunity through antibody secretion	
	Natural killer cells	Removal of tumor and virus-infected cells	

## Abbreviations

ADP	adenosine diphosphate;
ATP	adenosine triphosphate;
EGF	epidermal growth factor;
GDP	guanosine diphosphate;
GTP	guanosine triphosphate;
MMP	matrix metalloproteinase;
NO	nitric oxide;
PAF	platelet activating factor;
PAI-1	plasminogen activator inhibitor-1;
PDGF	platelet derived growth factor;
TFPI	tissue factor pathway inhibitor;
TGF-β	transforming growth factor β;
VEGF	vascular endothelial growth factor;
VWF	von Willebrand factor.
